# Epigenetics and Mechanobiology in Heart Development and Congenital Heart Disease

**DOI:** 10.3390/diseases7030052

**Published:** 2019-09-01

**Authors:** Dillon K. Jarrell, Mallory L. Lennon, Jeffrey G. Jacot

**Affiliations:** 1 Department of Bioengineering, University of Colorado Anschutz Medical Campus, Aurora, CO 80045, USA; 2 Department of Pediatrics, Children’s Hospital Colorado, Aurora, CO 80045, USA

**Keywords:** DNA methylation, histone modification, microRNA, cardiac development, congenital heart defects, endocardium, hemodynamics, mechanotransduction, biomarkers, maternal diabetes

## Abstract

Congenital heart disease (CHD) is the most common birth defect worldwide and the number one killer of live-born infants in the United States. Heart development occurs early in embryogenesis and involves complex interactions between multiple cell populations, limiting the understanding and consequent treatment of CHD. Furthermore, genome sequencing has largely failed to predict or yield therapeutics for CHD. In addition to the underlying genome, epigenetics and mechanobiology both drive heart development. A growing body of evidence implicates the aberrant regulation of these two extra-genomic systems in the pathogenesis of CHD. In this review, we describe the stages of human heart development and the heart defects known to manifest at each stage. Next, we discuss the distinct and overlapping roles of epigenetics and mechanobiology in normal development and in the pathogenesis of CHD. Finally, we highlight recent advances in the identification of novel epigenetic biomarkers and environmental risk factors that may be useful for improved diagnosis and further elucidation of CHD etiology.

## 1. Introduction

Congenital heart disease (CHD) is characterized by improper heart formation manifesting as defects involving the heart walls, valves, or blood vessels. Approximately 1% of infants are born with CHD, and 25% of these require surgery within one year of birth [1]. The prevalence and lethality of CHDs have warranted extensive research for several decades, but the etiology of most cases remains unknown. Despite monumental advances in our understanding of heart development, its staggering complexity has continued to limit our ability to identify and target the molecular mechanisms that underlie CHD. Heart development is controlled by several overlapping morphogenic systems that regulate intricate cardiac transcriptional networks. Nodal, bone morphogenic proteins (BMPs), Wnts, Sonic hedgehog, NOTCH, neuregulin, retinoic acid, fibroblast growth factors (FGFs), and other signaling molecules are known to play essential roles in regulating cardiogenesis, primarily by controlling cardiac transcription factors MESP1, GATA4, NKX2.5, HAND1, HAND2, ISL1, and several TBX transcription factors [2,3,4,5,6,7,8,9]. Single mutations in specific morphogens, their target transcription factors, or downstream cardiac genes have been shown to be independently sufficient to cause ~10% of CHD; however, for the majority of cases, the cause remains unknown [10,11,12,13,14,15,16,17,18]. To advance the diagnosis, understanding, and treatment of CHD, the extra-genomic factors critical for heart development need to be uncovered.

Epigenetics and mechanobiology are two extra-genomic mechanisms that are capable of regulating gene expression. Epigenetics involves the control of transcription and translation without changing the underlying nucleotide sequence. Epigenetic modifications can be inherited by daughter cells during cell division and include DNA methylation and histone modifications, which control gene accessibility, and non-coding RNAs, which primarily control mRNA translation. An increasing body of clinical and experimental research has identified aberrant epigenetic patterns in several polygenic diseases, including CHD [19]. Mechanical forces and the proper sensing of these forces by cells is also critical for heart development. Morphogenesis of heart structure occurs in tandem with continuous contraction and dynamic changes in blood flow. In the absence of flow, the heart does not develop properly, and disturbing normal blood flow leads to a variety of cardiac malformations [20,21]. Research in mechanobiology has identified a number of mechanosensitive pathways that regulate cardiac development, and epigenetic modifiers have also been implicated as key regulatory components of flow-responsive signaling pathways. This review aims to summarize the current understanding of heart development and the extra-genomic systems that, when perturbed, can cause CHD.

## 2. Stages of Heart Development and Manifestations of Congenital Heart Disease

Heart development is uniquely complex because it is the first organ to develop, it is strikingly asymmetric, it arises from four distinct progenitor cell sources, and it is subject to strong and changing mechanical forces. In addition to its clear organ-level asymmetry, the heart also exhibits tissue-level asymmetry in that it is not comprised of repeating functional units like the pancreas (islets), kidney (nephrons), or lungs (alveoli). This section describes the stages of human heart development and discusses the defects that manifest at each stage. Multiple excellent reviews describe the molecular biology of heart development in further detail [9,22,23,24].

### 2.1. Gastrulation and the Two Heart Fields

**Figure 1:**
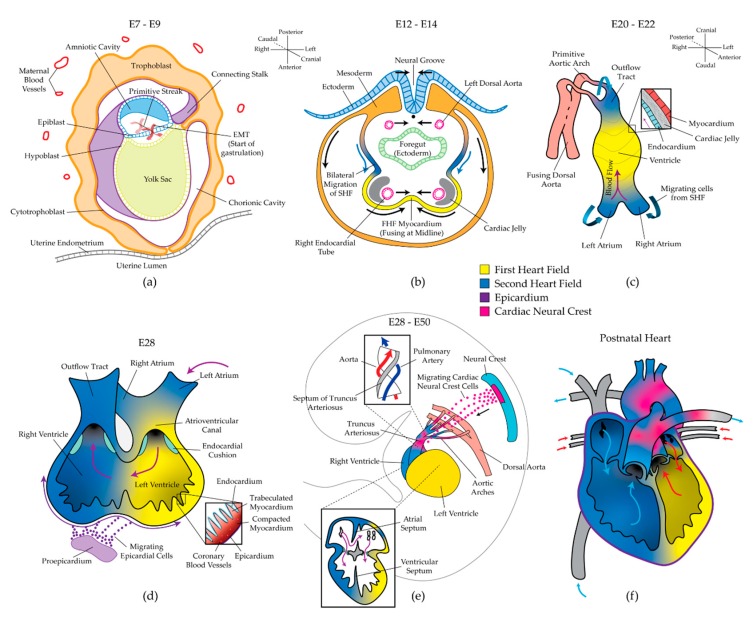
Human heart development. (**a**) The implanted blastocyst quickly forms the bilaminar embryo. The primitive streak forms in the epiblast, and an epithelial-to-mesenchymal transition (EMT) near the streak causes gastrulation to begin. (**b**) Bilateral migration of the first heart field (FHF) and second heart field (SHF) yields the cardiac crescent and endocardial tubes. (**c**) The bilateral cardiogenic regions fuse to form the bilaminar linear heart tube. SHF cells migrate into the poles, and the primitive outflow tract (OFT) begins to fuse with the dorsal aorta. (**d**) Asymmetry is broken with leftward cardiac looping. The proepicardium undergoes EMT to form the epicardium. Inset: trabeculation, compaction, and formation of the primitive coronary vasculature. (**e**) Neural crest cells migrate to populate the OFTs. Insets: Septa separate the truncus arteriosus and the left and right heart; the endocardial cushions give rise to the four valves. (**f**) The postnatal human heart.

Between human embryonic days 6 and 9 (E6–E9), the blastocyst migrates through the uterine epithelium into the uterine mucosa. The inner cell mass of the blastocyst gives rise to the bilaminar embryo, consisting of the epiblast and hypoblast. The hypoblast contributes primarily to the anterior yolk sac, while the epiblast further differentiates to form the prechordal plate and posterior amniotic sac. From E9–E10, gastrulation begins as the primitive epiblast undergoes epithelial-to-mesenchymal transition (EMT) at a furrow called the primitive streak (Figure 1a). Subjacent sheets of endodermal and mesodermal cells emerge through the streak, forming the trilaminar embryo. From E12–E15, two subsequent populations of cardiac mesoderm migrate bilaterally away from the streak and coalesce at the ventral midline of the embryo (Figure 1b) [25,26]. These two distinct progenitor populations have been termed the first heart field (FHF) and second heart field (SHF). Several lineage-tracing experiments have demonstrated that the FHF gives rise to the left ventricle (LV) and parts of the left atrium, while the SHF populates the entire right heart, parts of the left atrium, the majority of the outflow tracts (OFTs), and the proepicardium (Figure 1f) [25,27,28]. This discovery has led to the hypothesis that the two heart fields can be independently dysregulated, causing chamber-specific CHDs such as hypoplastic left heart syndrome (HLHS) or DiGeorge Syndrome [29]. While some markers of the two heart fields have been identified [30,31], improved phenotyping of the heart fields and the cells they give rise to will allow for better testing of this hypothesis. During the bilateral migration of the heart fields, angiogenic cords form within the cardiogenic areas and give rise to two endocardial heart tubes in the crescent by E18 (Figure 1b) [32]. Improper fusion of the two bilateral regions results in cardia bifida, an embryonic-lethal phenotype in which cardiac development proceeds independently in two distinct areas.

### 2.2. Formation of the Linear Heart Tube

From E20–E22, the endocardial tubes fuse to form the primary linear heart tube, consisting of an inner layer of endocardium and an outer layer of myocardium (Figure 1c). The endocardium functions as a layer of vascular endothelium and is also essential for the development of the coronary vasculature [33], cardiomyocyte proliferation [34,35], chamber septation [36], and formation of the heart valves [37]. The endocardium and myocardium are separated by a layer of extracellular matrix (ECM) proteins and polysaccharides called cardiac jelly. Although cardiac jelly was once thought to be inert, it has recently been demonstrated to be essential for proper pumping mechanics, endocardial–myocardial crosstalk, and the emergence of cardiac trabeculae and valves [38,39]. For example, a recent study found that the non-uniform distribution of cardiac jelly is necessary for efficient valveless pumping, and its specific fiber architecture powers the refilling of the early ventricle [40]. The heart tube initially elongates linearly as new myocardial, endocardial, and smooth muscle cells are added to the two poles of the tube from the SHF (Figure 1c). Simultaneously, two endothelial tubes posterior to the heart fields fuse to form the dorsal aorta. The cranial pole of the heart tube gives rise to the OFT, from which six pairs of vessels emerge and anastomose with the dorsal aorta. At this time, contraction begins. Interestingly, the precise mechanism of pumping that achieves unidirectional valveless flow remains controversial. Initially, the tubular heart was thought to function as a peristaltic pump; however, this convention has been recently challenged with an impedance (Liebau) pump model. These conflicting hypotheses have been excellently reviewed [41]. In either case, the start of contraction and resulting mechanical forces play an immediate and sustained role in heart development.

### 2.3. Cardiac Looping and Chamber Specification

During the third and fourth weeks of human development, overt left–right symmetry is broken as the linear heart tube undergoes rightward looping. The poles of the tube continue to grow linearly; however, the middle of the tube undergoes radial growth as a single ventricle “balloons” outward [42]. Four heart regions become distinguishable by E28: the atrium, atrioventricular canal (AVC), ventricle, and OFT (Figure 1d). The dysregulation of left–right asymmetry at this stage of heart development yields severe disease phenotypes, including heterotaxy syndrome, transposition of the great arteries (ToGA), double outlet right ventricle (DORV), and double outlet left ventricle (DOLV). Several ideas have been proposed to explain the mechanisms that initiate and maintain left–right asymmetry in heart development, but these mechanisms remain poorly understood [43]. Concurrent with cardiac looping, the proepicardium undergoes EMT, and a third progenitor population migrates to form an epithelial cell layer around the looping myocardium (Figure 1d). The resultant epicardium contributes vascular cells and fibroblasts to the myocardium, stimulates myocyte proliferation in a paracrine fashion, and has recently been suggested to give rise to additional cardiomyocytes during development and heart repair [44,45].

### 2.4. Septation and Formation of the Valves

Cardiac looping and chamber specification are completed by approximately E28. However, the heart does not reach its final recognizable form until approximately E50. The three final morphologic events that occur in this span are: (1) septation, which separates the four heart chambers and divides the truncus arteriosus into the aorta and pulmonary artery, (2) the formation and fusion of the pulmonary veins and vena cava with the left and right atria, respectively, and (3) the formation of the four cardiac valves (Figure 1e). These morphogenic events have been previously reviewed in detail [36]. Briefly, atrioventricular (AV) cushions composed of endocardial-derived mesenchymal cells and cardiac jelly span the AV canal and give rise to the mitral and tricuspid valves. Muscular septa emerge from the atrial roof and ventricular floor and grow toward the AV cushions. The ventricular septum fuses with the AV cushions, but the atrial septum remains unattached to form the foramen ovale, which limits pulmonary blood flow prior to birth. The separation of the truncus arteriosus into the aorta and pulmonary artery is driven largely by a fourth cardiac progenitor population that migrates from the neural crest (Figure 1e) [46].

Most complex cases of CHD manifest during these final three morphologic stages. Improper septation of the chambers yields atrial septal defects (ASD) and ventricular septal defects (VSD), which occur in isolation or in combination with other malformations as in tetralogy of Fallot (TOF). Improper septation of the truncus arteriosus can yield DOLV or DORV, ToGA, persistent truncus arteriosus, and coarctation of the aorta. Improper fusion of the pulmonary veins with the left atrium has been broadly termed “anomalous pulmonary venous return” and largely includes cases in which the veins empty into the right atrium rather than the left. Valve malformations include the complete absence of valves (as in tricuspid atresia), stenosed or narrowed valves (as in pulmonary stenosis), and incorrect leaflet geometries (as with bicuspid aortic valves). Malformed septa, valves, and blood vessels can dramatically alter the mechanical environment in adjacent chambers during cardiac maturation, leading to pathogenic remodeling and syndromic effects.

### 2.5. Cardiac Maturation

In addition to the complex morphological changes described so far, the transformation of the developing heart from a smooth, bilaminar tube to the mature vertebrate heart requires extensive myocardial growth. This process can be summarized in three steps: trabeculation, compaction, and thickening of the compact myocardium. Several comprehensive reviews have described these processes and the related development of the coronary vasculature [47,48,49,50,51]. After cardiac looping, myocardial protrusions called trabeculae extend into the heart lumen (Figure 1d, inset). Trabeculae increase the surface area of the heart wall to allow for adequate nutrient diffusion prior to the formation of the coronary vasculature and are also involved in the development of the conduction system [52,53]. During myocardial compaction, the trabeculae cease luminal growth and begin thickening radially. As the bases of the trabeculae coalesce, the layer of compact myocardium thickens in conjunction with the formation of coronary vessels (Figure 1d, inset). Further thickening of the compact myocardium is driven by cardiomyocyte proliferation and hypertrophy. Cardiomyocyte proliferation remains the subject of intense research because of its implications in regenerative medicine and tissue engineering [54,55]. Impaired trabeculation, compaction, or wall thickening are often related to improper cardiomyocyte proliferation and maturation, and result in severe phenotypes, including left ventricular non-compaction (LVNC) and HLHS [56].

The signaling molecules known to regulate cardiomyocyte proliferation and maturation include endocardial-derived neuregulin and endothelin, epicardial-derived FGFs, retinoic acid, and platelet-derived growth factor (PDGF), insulin-like growth factor (IGF), Wnts, NOTCH, reactive oxygen species, and ECM components [57,58,59,60,61,62,63,64,65,66]. However, our understanding of the regulation of cardiomyocytes and other heart cell types remains incomplete. Epigenetic and mechanical pathways are emerging as essential regulators of cardiac differentiation, proliferation, and maturation. In the following sections, these pathways will be further defined and discussed in the context of normal heart development and CHD.

## 3. Epigenetics and Congenital Heart Disease

**Figure 2:**
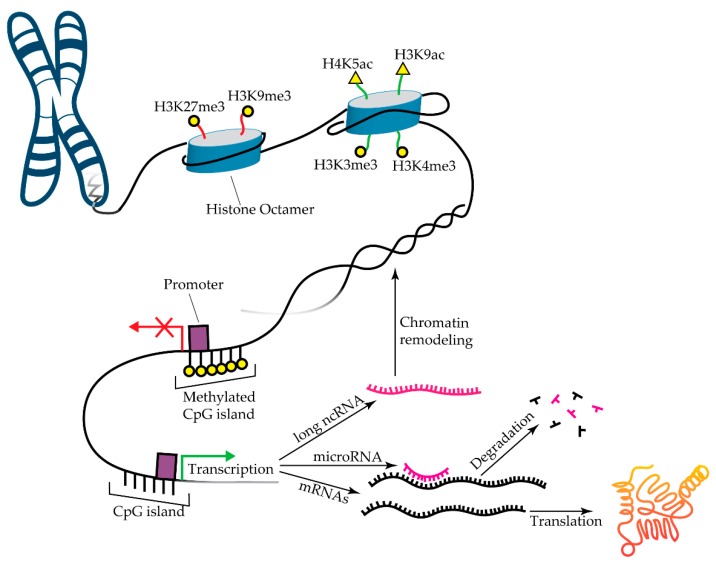
Mechanisms of Epigenetic Modification. Representative histone marks are depicted, but there are several other sites of histone methylation and acetylation. By altering chromatin structure, H3K27me3 and H3K9me3 inhibit transcription, while H4K5ac, H3K9ac, H3K3me3, and H3K4me3 promote transcription. Figure inspired by [67,68]. Reproduced with permission from D’Addario, et al., FEBS Journal; John Wiley and Sons, 2013 and Joosten, et al., Nature Reviews Urology; Springer Nature, 2018.

Epigenetics refers to the set of mechanisms that control gene expression beyond the nucleotide sequence of the genome. Three canonical mechanisms of epigenetic regulation are DNA methylation, histone modifications, and non-coding RNA activity (Figure 2). Importantly, these mechanisms are heritable during cell division and thus play an essential role in differentiation and the maintenance of cell fate. Furthermore, the ability of single epigenetic modifications to control the expression of several genes has implicated their dysregulation in many polygenic diseases, including CHD. In addition, several studies have identified erroneous expression of epigenetic modifiers and/or abnormal epigenetic modifications in the context of CHD. These modifiers and modifications are discussed in detail in the following sections and are summarized in Table 1.

### 3.1. DNA Methylation

In DNA, sequential cytosine–guanine nucleotides in the 5′ to 3′ direction are called CpG dinucleotides and can be methylated at the 5 position of cytosine. CpG dinucleotides would be expected to make up ~4% of a random genome; however, only ~1% of the human genome is composed of CpG [69]. While CpG dinucleotides are underrepresented in the genome as a whole, they are enriched in CpG islands. CpG islands are 300–3000 base pair regions that tend to be non-methylated, in contrast with most CpG dinucleotides residing outside of islands [70]. Approximately 70% of gene promoters exist in CpG islands, and methylation of these areas corresponds with gene silencing [71]. In addition to this canonical mechanism, CpG islands have been identified outside of promoter regions, and methylation of these islands sometimes increases transcription [69]. Methods of DNA methylation analysis have been previously reviewed [72].

The spatiotemporal expression of cardiac genes during development is astonishingly fine-tuned, and DNA methylation plays a critical role. Cardiac DNA methylation has been compared across embryonic stages, between CHD and control tissues, and between neonatal and adult tissues. One study conducted genome-wide methylation analysis in embryonic mouse hearts at stages corresponding to chamber development and maturation [73]. In hearts isolated from E11.5–E14.5 embryos, the authors identified 79 genes with differential DNA methylation that correlated with altered expression. Many of the genes are involved in heart development; notably, Has2 (hyaluronan synthase 2) is required for formation of the epicardium, septa, and heart valves, and was found to be downregulated via DNA methyltransferase 3b-mediated enhancer methylation at E14.5 [73]. In another study, long non-coding RNA uc.167 (lncRNA uc.167) was found to control Mef2c expression via DNA methylation. Mef2c is a cardiac transcription factor that is essential for cardiomyocyte proliferation and sarcomere assembly, and lncRNA uc.167 overexpression results in decreased cardiomyocyte differentiation efficiency and increased cardiomyocyte apoptosis [74].

Studies comparing DNA methylation status in newborns with aortic valve stenosis (AVS), TOF, and VSDs have identified several aberrant patterns in diseased patients [75,76,77]. A total of 52 genes with significantly altered DNA methylation were identified in the blood of newborn AVS patients compared to newborn healthy infants. Of particular interest are APOA5 (a determinant of plasma triglyceride levels) and PCSK9 (an activator of low-density lipoprotein receptors), which were both hypermethylated and are both considered major risk factors for coronary artery disease in adults [75]. In myocardial biopsies from patients with TOF and VSD, hypermethylation was observed in the promoter region of SCO2, which is a cytochrome c oxidase protein. The authors hypothesized that the resultant SCO2 downregulation drives the metabolic state of cardiac cells toward glycolysis, delaying terminal differentiation and promoting cardiomyopathy and heart failure [76]. In another study assessing the DNA methylation of key cardiac transcription factors, Nkx2.5 and chamber-specific Hand1 were found to be hypermethylated in patients with TOF [77]. As expected, the expression of these two transcription factors was found to be downregulated. In a revealing study, monozygotic twins discordant for DORV were analyzed for genomic and epigenomic differences [78]. Not surprisingly, very few genetic differences were identified. However, 121 transcription factor binding sites were differentially methylated, including the hypermethylation of ZIC3 (a zinc finger protein involved in Wnt signaling) and NR2F2 (a protein important in heart development), in the diseased twin. This finding highlights the importance of epigenetics in diseases with complex etiologies, such as CHD.

Another study analyzed the DNA methylome of cardiomyocytes isolated from newborn, adult, and adult failing hearts [116]. Most notably, the authors found that DNA methylation, mediated by DNA methyltransferases 3A and 3B, explains the prenatal–postnatal switch observed in several fetal sarcomeric proteins. In addition, the authors found that cardiomyocyte DNA methylation in human adult failing hearts as well as in murine left ventricular-overload hearts resembled a fetal methylation state. Collectively, these studies demonstrate that DNA methylation is tightly regulated during cardiac differentiation and maturation. Advancing our understanding of these spatiotemporal patterns may be insightful for the treatment of CHD and the pathologic remodeling that often leads to heart failure in CHD patients.

### 3.2. Histone Modification

Nucleosomes—histone octamers tightly wrapped by DNA—are the fundamental unit of chromatin and are necessary for the compact storage of DNA in the nucleus. However, this arrangement renders DNA largely inaccessible to the transcription factors and initiation complexes required for gene expression. Histone post-translational modifications (hPTM) effectively alter chromatin architecture, allowing for the control of transcription (Figure 2). hPTM primarily include methylation and acetylation, but can also include ubiquitination, phosphorylation, and sumoylation. The nomenclature for histone modifications has three sequential parts: (1) the specific histone protein (H2A, H3, etc.), (2) the amino acid type and location (K4, K27, etc.), and (3) the type of modification (me3, ac, etc.). For example, a trimethylation at lysine 27 of histone 3 is written as H3K27me3. Methods for hPTM analysis have been previously reviewed [117].

Histone-modifying proteins and complexes generally associate with transcription factors to regulate gene transcription at specific sequences of regulatory DNA. Several histone modifiers have been shown to be essential for cardiac development through conventional knockout studies [79,83,91,92,93,97,98,118]. In addition, aberrant patterns of hPTM have been associated with CHD, and known regulators of heart development have been proven to function via hPTM. For example, Wolf–Hirschhorn syndrome (WHS) is a congenital condition characterized by delayed growth and underdevelopment of several organ systems, including the heart. The gene encoding Wolf–Hirschhorn candidate protein 1 (WHSC1), an H3K36me3-specific methyltransferase, is deleted in every known clinical case of WHS. One study generated a murine Whsc1-knockout to elucidate the pathogenic role of Whsc1 deletion [79]. The researchers found that Whcp1 associates with several transcription factors, including Nkx2.5, and activates transcription at transcription factor target sites. Similarly, two consecutive studies revealed that JARID2/Jumonji functions via interaction with the histone methyltransferase SETDB1. The deletion of Jarid2 in mice resulted in VSD, DORV, and hypertrabeculation reminiscent of impaired ventricular compaction [80,118]. Molecularly, the knockout prevented the accumulation of SETDB1 and H3K9me3 at JARID2 gene targets, most notably the Notch1 promoter in the endocardium [80].

#### 3.2.1. Histone Deacetylases in CHD

Histone acetylation promotes transcription by loosening the tight interaction of DNA with histones. Therefore, histone deacetylase enzymes (HDACs) are associated with gene silencing. In cardiac development, our growing understanding of transcription factors has significantly outpaced that of the associated chromatin modifiers. Two studies recently addressed this discrepancy by demonstrating that HDAC2 and HDAC3 associate with the central cardiac transcription factors GATA4 and TBX5, respectively [81,82]. The resultant downregulation of TBX5 target genes is essential for proper cardiac differentiation and cardiomyocyte proliferation. A protein interaction study demonstrated that the lysine methyltransferase G9a interacts with MEF2 and HDAC5 and prevents the export of HDAC5 from the nucleus. MEF2 target genes, including several that encode sarcomeric proteins, are effectively downregulated in the presence of G9a [83]. SMyD1/m-Bop is a methyltransferase that is highly expressed in the SHF. Decades of research have found that SMyD1 can control gene expression by (1) repressing transcriptional activity via association with HDAC1-3 and NCoR/SMRT complexes [85], (2) activating transcriptional activity by catalyzing H3K4me3 [86], (3) localizing to muscle-specific promotors via association with the transcription factor skNAC1 [85], or (4) catalyzing post-translational modifications on ER stress proteins, which are necessary for cardiomyocyte proliferation and survival [87].

A recent study corroborated these findings by further demonstrating the over-activity of HDACs in single ventricle (SV) CHD [119]. The authors suggested that HDAC inhibitors be investigated for the treatment of CHD. HDAC inhibitors are currently FDA approved for oncology applications. Preclinical studies have yielded promising results for their use in treating adult heart failure, finding a reduction in cardiac hypertrophy and fibrosis while preventing reversion to fetal cardiac gene programs [120]. Future studies need to investigate HDAC inhibitors for the treatment of CHD, particularly in complex cases prone to pathologic remodeling.

#### 3.2.2. Polycomb Group Proteins in CHD

Trimethylation of H3K27 drives robust gene silencing, even in the presence of non-methylated DNA [116]. Polycomb group (PcG) proteins, including the polycomb repressive complexes (PRC1 and PRC2) are the fundamental mediators of H3K27 methylation. The dysregulation of PcG proteins and aberrant H3K27me3 yield critical CHD phenotypes. The cardiac-specific deletion of Ezh2, the methyltransferase enzyme in PRC2, yielded mouse hearts with VSD, ASD, hypoplastic endocardial cushions, impaired EMT, and increased cardiomyocyte apoptosis [88]. The developmental proteins TBX2 and Hey2 were both downregulated in mutant hearts, likely because a downregulator of these genes failed to be methylated in the absence of Ezh2. A similar expression profile was identified in an epigenetic analysis of induced pluripotent stem cell-derived cardiomyocytes (iPSC-CM) from patients with HLHS or normal biventricles [90]. The researchers found that TBX2, Hey2/NOTCH1, Nkx2.5, and Hand1 gene expression was downregulated in HLHS iPSC-CM compared to control iPSC-CM. Increased H3K27me3 and decreased H3K4me2 were observed at these genes (Figure 2). This study was one of the first to demonstrate the patient-specific potential that iPSC-CM possess for in vitro CHD modeling and the study of epigenetics.

There are also enzymes that catalyze the removal of H3K27me3 and reactivate gene expression. UTX and JMJD3 are both capable of this catalysis and are both highly expressed in the developing embryo. Furthermore, both are required for the differentiation of cardiac cells [91,92]. JMJD3 appears to be essential for the earliest stages of differentiation, as it upregulates the expression of the mesoderm marker Brachyury (T) and Wnt pathway-mediator β-catenin [91]. Both enzymes upregulate GATA4 expression, and UTX also maintains the expression of NKX2.5 and TBX5 throughout heart development [92]. Impaired differentiation at these stages typically results in CHD or embryonic lethality.

#### 3.2.3. Trithorax Group Proteins in CHD

Trithorax group (TrxG) proteins represent the second fundamental family of histone methyltransferases. In contrast with PcG proteins, TrxG proteins primarily catalyze H3K4 methylation. Similarly to PcG proteins, several studies have demonstrated that their dysregulation yields CHD. In a knockout study, the TrxG protein MLL2 was found to be essential for the earliest stages of cardiac differentiation from mesoderm, primarily by upregulating core cardiac transcription factors NKX2.5, TBX5, and MEF2c via H3K4me3 [93]. Also in the TrxG are SWI/SNF chromatin remodeling complexes, which include the Brg/Brm-associated factor (BAF) complexes. Two elegant studies demonstrated that slight perturbations in the dosing of BAF proteins result in severe CHD phenotypes [95,96]. An intermediate reduction in Baf60c severely impaired OFT development [95]. Disruption of the balance between Brg1 and the cardiac transcription factors NKX2.5, TBX5, and TBX20 also yielded severe ventricular defects in mice [96]. In another study that connected known transcription factor functions with epigenetics, TBX1 was shown to interact with the BAF complex to regulate Wnt5a expression. Wnt5a is expressed predominantly in the SHF, and the insufficient expression of TBX1 or Wnt5a yield hypoplastic right heart phenotypes [97]. In addition to transcription factors and histone modifiers, BAF complexes interact with tissue-specific proteins that recruit the complex to specific histone marks. DPF3 recruits the BAF complex to several post-translational marks on histones 3 and 4, and its deletion results in impaired cardiac looping and muscle fiber assembly [98]. The ability of histone modifiers to alter cardiac transcription factor activity, particularly in the absence of any detectable gene mutation, makes them strong candidates for CHD etiology and therapeutic targeting.

### 3.3. Non-Coding RNA

Non-coding RNAs involved in the control of gene expression primarily include long non-coding RNA (lncRNA) and microRNA (miRNA or miR). lncRNAs can directly interact with chromatin remodeling complexes to further modulate transcription [121], while miRNAs primarily interact with mRNA transcripts to repress translation. Humans express over 1000 miRNAs that collectively regulate ~30% of genes [122]. Our understanding of their role in cardiac development has advanced significantly in the last two decades, and their association with CHD continues to be uncovered. Methods for the analysis of miRNA have been previously reviewed [123].

miRNAs are often tissue-specific and tend to regulate gene expression by modulating feedback loops. A pair of studies found that miR-1-1 and miR-1-2 are expressed specifically in cardiac and skeletal muscle progenitor cells [99,101]. Further investigation by these studies and several others demonstrated that Mef2 upregulates miR-1-1 and miR-1-2 expression, which suppress Hand2 and class II HDAC translation [99,101,102]. Downregulated HDAC4 upregulates Mef2 in a positive feedback loop, and the control of Hand2 maintains a careful balance between cardiac cell differentiation and expansion [103]. Furthermore, specific doses of miR-1-1 and miR-1-2 were shown to have profound effects in animal models; slightly decreased expression caused impaired cardiac morphogenesis, cell cycle regulation, and development of the conduction system in mice [101]. However, miRNAs are not always tissue-specific. A traditional murine knockout experiment revealed that deletion of miR-17~92, a cluster of six miRNAs, resulted in VSDs, lung hypoplasia, and impaired B cell development [104].

#### miRNA Profiles in CHD Patient Biopsies

Several studies have compared the miRNA expression profiles in patients with and without specific CHDs [100,109,110,111,112,113,114]. One study compared the miRNA profiles from right ventricle biopsies taken from HLHS patients after heart transplant and from non-failing donor hearts [109]. The researchers identified 13 differentially regulated miRNAs. The expression levels of three of the 13 miRNAs (miR-99, miR-100, and miR-145) returned to normal after palliative surgery, suggesting that volumetric loading can regulate gene expression via miRNAs. The chromatin remodeling protein BAZ2A was also found to be regulated by these three miRNAs (Table 1), but its role in HLHS, hypertrophy, and heart failure remains to be elucidated. Notably, miR-204 was found to be upregulated in both HLHS and pediatric idiopathic dilated cardiomyopathy patients [108], making it a promising therapeutic target for multiple forms of pediatric heart failure. A recent study was the first to compare the circulating miRNA profiles of CHD patients compared to healthy controls. Eight miRNAs were differentially expressed in patients with VSDs, including several known to regulate genes important for RV morphogenesis [111]. Another study found that miR-30 and miR-133 directly suppress connective tissue growth factor (CTGF), a pro-fibrotic factor that has been shown to be elevated in cases of adult pathologic LV hypertrophy [110]. As expected, miR-30 and miR-133 were both downregulated in diseased fibrotic tissues. Furthermore, the overexpression of these two miRNAs reduced the proliferative capacity of cardiomyocytes and fibroblasts in vitro. CTGF is also regulated by the Hippo–YAP pathway, which is an area of interest in cardiac tissue engineering because of its ability to modulate cardiomyocyte proliferation [124]. While the role of these miRNAs in pediatric heart disease remains to be uncovered, they could represent a promising knockdown target for cardiomyocyte regeneration.

Multiple studies have identified irregular miRNA expression in biopsies from TOF patients [100,112,113,114,125]. One such study identified 44 cardiac genes whose expression was significantly different in TOF myocardium compared to normal myocardium. The expression of these 44 genes was negatively correlated with that of 33 miRNAs [125]. Further investigation of the 33 miRNAs revealed miR-421 as a regulator of SOX4, which is essential for proper Notch signaling and OFT development [113]. An independent study identified 18 miRNAs with significantly different expression in TOF myocardium. Specifically, miR-424 was found to be overexpressed, resulting in increased cardiomyocyte proliferation and decreased HAS2 and NF1 expression in the right ventricular OFT [112]. A third study identified miR-940 as the most downregulated of 75 dysregulated miRNAs in TOF tissues [114]. miR-940 is primarily expressed in cardiac OFT relative to other cardiac chambers and its reduction increases cardiomyocyte proliferation. Furthermore, it was found that miR-940 regulates JARID2, a chromatin modifier essential for proper Notch signaling and heart development [114]. A fourth study analyzed the expression of connexin-43 (Cx43) in TOF myocardium and sought to identify miRNAs that may contribute to its dysregulation [100]. Cx43 was upregulated in diseased tissue, and miR-1 and miR-206 were downregulated. However, the causality between miR-1/miR-206 and Cx43 and between Cx43 and TOF requires future work to be elucidated.

miRNA therapies for CHD are particularly attractive compared to other epigenetic pathways because of the vast arsenal of genes that they regulate, as well as their gene and tissue specificity. Furthermore, miRNAs have been shown to be essential for cardiomyocyte proliferation, maturation, and pathogenic remodeling, making them promising candidates for the preservation and correction of heart function in patients with structural heart defects.

## 4. Mechanobiology and Cardiac Development

Mechanobiology is the study of how cells sense and respond to biomechanical stimuli. This process is critically important during cardiac development. When hemodynamic conditions are intentionally disrupted in animal models, defects resembling those found in patients with CHD arise. The various surgical interventions used to alter hemodynamics in animal models and the associated cardiac malformations that emerge have been previously reviewed [126]. This section will review the role of hemodynamic forces in cardiac morphogenesis and the mechanosensitive signaling pathways involved in this complex developmental process.

### 4.1. Mechanosensitive Pathways in Chamber Development and Trabeculation

**Figure 3:**
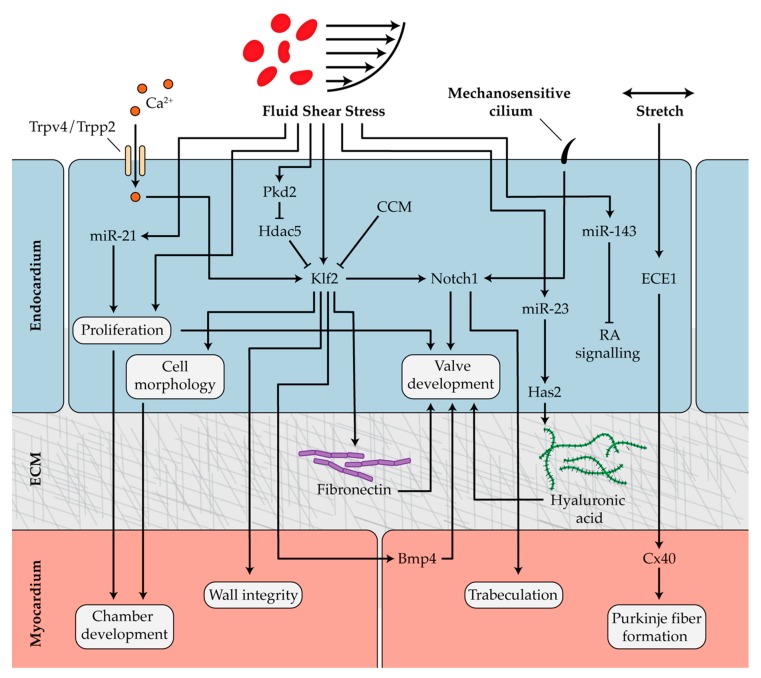
Mechanosensitive pathways involved in heart development. During chamber morphogenesis, hemodynamic forces control endocardial cell proliferation and, through Klf2a, direct endocardial cell morphology. Blood flow also has an impact on myocardial wall integrity, an effect that is mediated by *klf2* expression in the endocardium. Cilia sense fluid forces and activate Notch1 signaling to regulate trabeculation. During valve development, Klf2a responds to blood flow to activate Bmp4 in the myocardium and fibronectin synthesis in the extracellular matrix (ECM). Klf2a is positively regulated by mechanosensitive ion channels Trpv4 and Trpp2 and suppressed by cerebral cavernous malformation (CCM) proteins and the chromatin modifier Hdac5. Stretch induces endocardial endothelin converting enzyme 1 (ECE1) and myocardial Cx40 expression, which controls Purkinje fiber differentiation. Flow-responsive miR-21 regulates proliferation in the developing valves. Hyaluronan synthase 2 (Has2) produces hyaluronic acid, an important component of the ECM. Shear stress-responsive miR-23 regulates Has2 and is critical for valve formation. miR-143 inhibits retinoic acid (RA) signaling in a spatially-controlled manner during cardiogenesis.

Hemodynamic conditions in the developing heart play a critical role in regulating cardiac looping and trabecular patterning. One study in zebrafish found that blood flow and contractility independently regulate cell shape in the emerging ventricle, and both biomechanical forces are necessary for chamber formation [127]. Endocardial cells are critical for the proper development of cardiac chambers, and a lack of endocardium in zebrafish embryos results in ballooning defects [128,129]. As the cardiac chambers grow, endocardial cells proliferate and acquire unique morphological features depending on their location within the developing heart. The inhibition of blood flow in developing zebrafish limits endocardial cell proliferation and alters cell shape and area, illustrating that chamber formation is a blood flow-dependent process [130]. Expression levels of the hemodynamic-responsive transcription factor Krüppel-like factor 2a (Klf2a) were lower in abnormally-reduced flow conditions, and knockdown of *klf2a* caused a significant increase in endocardial cell surface area [130]. These results demonstrated that Klf2a is regulated by blood flow and is an important mediator of endocardial cell shape and size during cardiac ballooning. An additional study showed that zebrafish *klf2* mutants have compromised myocardial wall integrity [131]. The overexpression of *klf2*, which is expressed in the endocardium and not in the myocardium, rescued the abnormal phenotype and was sufficient to restore the integrity of the myocardial wall [131]. These studies show that the loss of Klf2 results in defects in both endocardial and myocardial cell behavior. This suggests that fluid forces trigger *klf2* expression in the endocardium, and subsequent endocardial–myocardial interactions coordinate cell shape and chamber dimensions to control development of the myocardial wall (Figure 3).

Normal blood flow is necessary for trabeculation. However, even in the presence of appropriate flow conditions, trabeculation does not occur in zebrafish mutants lacking an endocardium. This indicates that signaling from the endocardium to the myocardium, initiated by flow sensing in endocardial cells, is necessary for trabeculation [132]. One physical mechanism by which endocardial cells detect blood flow is via cardiac cilia. Mechanosensitive cilia are found on endocardial cells lining the atrium, cardiac cushions, and trabeculations in the developing ventricle. Without cilia, the compact myocardium develops abnormally [133]. One study demonstrated that primary cilia found on the ventricular endocardium activate endocardial *notch1b* expression in response to flow, which controls trabeculation through crosstalk with the myocardium [134]. Interestingly, Klf2a activates Notch1b in response to blood flow even in the absence of cardiac cilia; however, both functional cilia and Klf2a are necessary for trabeculation [134]. These findings were recently corroborated using light-sheet microscopy to visualize developing trabeculae. This study confirmed that endocardial cells sense and respond to shear stress and upregulate Notch signaling to initiate trabeculation (Figure 3) [135].

### 4.2. Hemodynamics in Endocardial Cushion and Valve Formation

Endocardial cushions are specialized structures within the AVC that remodel to form the cardiac valves. Zebrafish mutants lacking a heartbeat do not form cardiac cushions [136]. Furthermore, restricting blood flow at either the inflow or OFT results in abnormal valvulogenesis [20]. These studies illustrate the importance of biomechanical forces in proper valve development. Prior to valve formation, the magnitude of retrograde, or reversing, blood flow peaks in the AVC [137]. Endocardial cells sense and respond to the shear stress generated by these complex flow patterns, which drives development of the cardiac cushions. Rather than migrating against the mean direction of flow, as endothelial cells do during vascular remodeling, endocardial cells converge in response to dynamic wall shear stress patterns generated from oscillatory flows in the AVC [138,139]. In addition to its role as a regulator of myocardial wall development and trabeculation, it was discovered that *klf2a* is also required for valve formation. The expression of *klf2a* and its downstream target *notch1b* become restricted to endocardial cells in areas of retrograde flow in the AVC [137]. Interestingly, *klf2a* was found to affect the levels of *bmp4* expression in the myocardium, providing evidence that tightly regulated crosstalk between the endocardium and myocardium is essential for valve development [137]. Fibronectin deposition in the AVC extracellular matrix is also regulated by Klf2a. Zebrafish mutants lacking expression of *klf2a* exhibit impaired fibronectin synthesis and cell recruitment to the AVC, both of which are necessary for valvulogenesis [140]. These results suggest that flow-dependent changes in mechanical properties within the endocardial extracellular environment may direct cell behavior and valve morphogenesis (Figure 3). In addition to defects in ventricular trabeculation, abnormal cardiac cushion development was observed in mice lacking cardiac cilia [133]. These results suggest that cilia-mediated flow sensing is an integral part of cardiac morphogenesis, including valve formation. However, additional studies are required to elucidate the exact mechanisms for the role of cilia in cardiac cushion and valve development.

Cerebral cavernous malformation (CCM) proteins are important regulators of the blood flow-dependent expression of *klf2a* in the developing zebrafish valve [141,142,143,144]. As *klf2a* expression increases, *heg1* expression is upregulated. Heg1 stabilizes the CCM protein Krit1, which subsequently represses *klf2a*. Thus, a negative feedback loop is established where Heg1 and Krit1 act to diminish levels of *klf2a* and its downstream target *notch1b* [145]. Reduced *notch1b* expression is known to be required for endocardial cushion cell sprouting, which is an important step in sculpting valve leaflets [140]. Knockdown of CCM proteins in zebrafish causes *klf2a* and *notch1b* to be overexpressed throughout the endocardium, which inhibits proper valve development. These results suggest that CCM proteins selectively reduce *klf2a* expression in a subset of endocardial cells, desensitizing them to blood flow, lowering their expression of *notch1b*, and coordinating the remodeling of cardiac cushions into valve leaflets [145]. Klf2a is also regulated by mechanosensitive calcium channels Trpp2 and Trpv4 in response to oscillatory flow in the AVC (Figure 3) [146].

### 4.3. Mechanotransduction During Formation of the Outflow Tract, Conduction System, and Epicardium

Aside from its presence in the AVC, elevated levels of klf2a and notch1b have also been found localized to the developing OFT [137]. A recent study has shown that contractile and hemodynamic forces play independent roles in OFT valve development [147]. Artificially increasing myocardial contractility in the developing zebrafish embryo resulted in valve hyperplasia, while inhibiting contractility prevented valve formation. Increasing wall shear stress alone also increased endocardial Notch1b activity in the OFT and led to valve hyperplasia. While these findings suggest that both wall shear stress and myocardial contractility are necessary, the relative importance of these two forces remains to be determined [147]. It has been established that flow is required for valve formation in the OFT; however, the mechanism of flow sensing in the OFT remains unclear [148]. Trpp2 and Trpv4 channels, which regulate flow sensing in valve development in the AVC and are involved in mechanosensing in the vasculature, may be promising candidates as mechanosensors in the OFT as well [146,149]. In addition, the Piezo family of ion channels, which are required for shear stress sensing in the vasculature, could detect similar forces in the developing OFT [150].

In chick embryos, endothelin converting enzyme 1 (ECE1) is expressed in the endocardium along sites where myocytes are recruited and differentiate into Purkinje fibers [151]. When chick embryos were treated with a stretch-sensitive ion channel antagonist, ECE1 was downregulated along with the Purkinje fiber marker Cx40. Furthermore, the expression of ECE1 and Cx40 is upregulated in pressure-overloaded hearts [152]. The inhibition of stretch mechanosensors delayed organization of the fibers, while pressure overload led to premature maturation of the Purkinje fibers. Taken together, the results suggest that biomechanical forces modulate ECE1 and Cx40 expression and are critical for the differentiation and patterning of Purkinje fibers (Figure 3) [152]. Hemodynamic stretch has also been implicated as an important coordinator of atrial conduction patterning. Mechanical loading of atrial muscle from chick embryos leads to the simultaneous induction of the conduction markers Cx40 and Nav1.5 as well as the cell cycle marker Cyclin D1. Loading initiates stretch-dependent expression of fast conduction markers and proliferation, ultimately regulating atrial conduction development [153]. Additional studies are needed to determine which cells sense and translate stretch in the atria and which mechanosensitive pathways are involved.

In addition to their localization to the endocardial surface, cilia are found on the epicardium [133]. In zebrafish, pericardial fluid flow generated by the heartbeat is necessary for the formation of the epicardium. These fluid forces cause the release of epicardial progenitors into the pericardial space where they adhere to the surface of the myocardium [154]. A recent study confirms that while the heartbeat alone is not required for proepicardial migration, it is important for proepicardial positioning and development of the epicardial layer [155].

### 4.4. Mechanical Regulation of Heart Epigenetics

Biomechanical forces are critical to the epigenetic mechanisms that regulate many stages of heart development. Epigenetic modifiers such as “mechano-miRs” and HDACs have been identified as key players in the mechanosensitive pathways that direct cardiac development. In zebrafish lacking a heartbeat, the expression of *miR-143* is absent and defects are observed in both the developing heart chambers and OFT [115]. In embryos with normal blood flow, a gradient of *miR-143* expression is established with high expression observed in the OFT, moderate expression in the ventricle, and low expression in the atrium. *miR-143* downregulates retinoic acid signaling in the endocardium [115]. In the absence of flow, the regional differences in *miR-143* expression and retinoic acid signaling are disrupted, leading to cardiac abnormalities during development (Figure 3). These results demonstrate an epigenetic link between blood flow and the pathways that control the spatial organization of the developing heart structures [115].

Epigenetic regulators also depend on cardiac mechanics during cardiac valve development. In zebrafish, *miR-21* is switched on and off by hemodynamic forces and its expression is localized to endocardium at constrictions of the AVC and OFT. Occluding blood flow resulted in abnormal expression of *miR-21* [106]. The rapid response of mechano-miRs was demonstrated by the fact that returning blood flow restored *miR-21* expression in as little as one hour. These findings identify *miR-21* as an essential component of a flow-dependent pathway that controls endocardial cell proliferation and the expression of genes required for valve development [106]. Has2 controls hyaluronic acid production and ECM composition in the developing heart. Has2-deficient mice fail to form cardiac cushions and ventricular trabeculae [156]. It has been shown in zebrafish that *miR-23* expression regulates *Has2* activity in endocardial cells and is necessary for valve formation (Figure 3) [107]. Although the exact mechanism remains unknown, it is possible that changes to the stiffness of the ECM, controlled by miR-23-regulated production of hyaluronic acid, generate biomechanical forces that are interpreted by endocardial cells to direct cushion and valve formation.

Valve development may also be controlled by mechanical influences on HDAC activity [84]. HDACs are inhibited by phosphorylation via members of the protein kinase D (PKD) family [157]. Zebrafish embryos with a mutation in *pkd2* have reduced levels of *klf2a* and *notch1b* in the endocardium and impaired valve development [84]. In the heart, PKD2 is known to phosphorylate HDAC5, which leads to its inactivation [84,157]. Without PKD2, the expression of downstream targets of HDAC5, including *klf2a* and *notch1b*, is diminished (Figure 3) [84]. This illustrates a key epigenetic component of the mechanotransduction pathways coordinating valve formation. Each of the aforementioned epigenetic regulators including miR-21, miR-143, and miR-23 as well as HDAC5 regulation of *klf2a* are also important hemodynamic-dependent mechanisms involved in vascular gene expression and function [158]. More research is needed in order to identify additional vascular epigenetic mechanisms that may also play a role in cardiac development. For example, miR-92a regulates *klf2a* expression in a flow-dependent manner in vascular endothelial cells and could be a potential regulator in heart development [159].

## 5. Recent Advances

### 5.1. Epigenetics as Biomarkers for CHD

Identifying epigenetic regulators as potential biomarkers of CHD could lead to earlier and more accurate diagnosis. Surprisingly, detection of prenatal CHD continues to be a challenge, with one study concluding that over 40% of CHD cases go undetected during pregnancy [160]. The diagnosis of CHD is largely dependent on fetal echocardiography, and there are currently no prenatal or postnatal biomarkers available for the detection of cardiac defects. Given that early diagnosis of CHD has been shown to greatly improve patient outcomes [161], there remains a need for the identification of accurate, non-invasive, and operator-independent biomarkers for prenatal diagnosis. Since epigenetic mechanisms play a crucial role in cardiac development, epigenetic regulators such as miRNAs and DNA methylation are being investigated as potential biomarkers for the diagnosis of CHD. A number of studies have identified differentially expressed miRNAs as potential biomarkers for the diagnosis of congenital heart defects [162]. An analysis of circulating miRNAs and miRNAs in cardiac tissue has shown altered miRNA expression in patients with a range of CHD including TOF, VSDs, and SV [111,162,163]. Significantly different expression of circular miRNA from VSD cardiac tissue were identified as a novel biomarker for this population [164]. Circular miRNAs are a distinct class of non-coding RNAs that regulate gene expression. The fact that they are more stable than linear RNA makes them a particularly attractive clinical biomarker and warrants further studies into these epigenetic regulators [165,166]. A recent study analyzing circulating miRNA levels in serum from pregnant women found a combination of four miRNAs that could distinguish CHD pregnancies from controls with high sensitivity and specificity. The discovery of these CHD-associated miRNAs in maternal serum makes them an attractive biomarker for prenatal diagnosis. A follow-up study in a larger cohort of patients is underway to validate the clinical potential of serum miRNAs for diagnosing fetal CHDs [167].

Distinct DNA methylation patterns have also been observed in patients with CHD and are being studied as potential biomarkers for CHD detection. CpG methylation levels were found to vary among various CHD types compared to controls, suggesting that these signatures could be used to discriminate between specific CHDs [168]. Using blood spot DNA from leucocytes, 25 differentially methylated CpG sites were found to be predictive of TOF, and many of the loci were localized to genes implicated in heart defect development [169]. Recent studies have also explored the use of placental tissue to identify methylation markers in CHD. Placental tissue is a currently underutilized and non-invasive source of potential biomarkers for the postnatal diagnosis of CHD. One study found 166 CpG loci with differential methylation in placental tissue from TOF [17]. Epigenetic analysis of placentas from patients with VSD revealed 80 CpGs and eight miRNAs that had the potential to accurately detect VSD [170]. Supporting the plausibility of these findings is the fact that many of the target genes associated with these differentially expressed epigenetic modifiers are known to be involved in several stages of cardiac development, including cardiac looping and chamber development [170].

**Figure 4:**
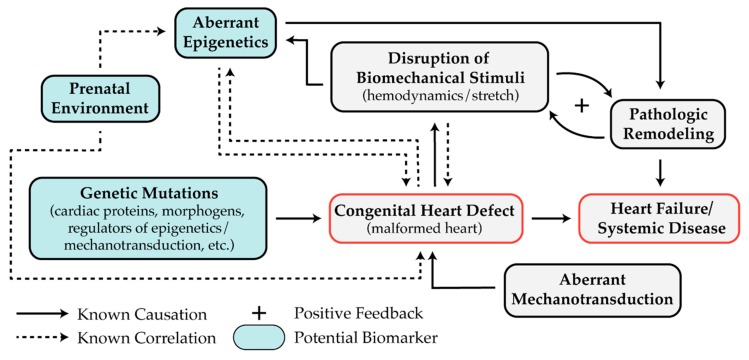
The disruption of highly synchronized regulators of gene expression leads to congenital heart disease. Crosstalk between genetics, mechanics, epigenetics, CHD, and the environment.

Differences in miRNA expression and DNA methylation may be valuable biomarkers for risk stratifying patients for CHD treatment (Figure 4). Further investigation of these promising epigenetic biomarkers will provide insight into the epigenetic mechanisms underlying the development and progression of CHD and may lead to future therapies targeting the epigenetic landscape.

### 5.2. Diabetes

Hyperglycemia caused by maternal diabetes mellitus during pregnancy is a well-known teratogen, and both type I and type II diabetes are significant risk factors for congenital heart defects [171]. Even in mothers without diabetes, elevated plasma glucose levels in the first trimester are associated with an increased risk of CHD [172]. A recent area of research seeks to understand whether epigenetic factors may be an underlying cause of diabetes-associated CHD. Dysregulated miRNAs have been identified in embryonic heart tissue from both type I and type II diabetic animal models compared to nondiabetic controls. Each of the target genes of the differentially expressed miRNAs are known to be involved in heart development [173,174]. A recent study compared DNA methylation signatures in buccal swab samples from infants with diabetic embryopathy and healthy infants who were exposed to diabetes in utero. Methylation levels at the *CACNA1C* locus were found to be drastically reduced in diabetes-exposed samples [175]. The expression of *CACNA1C*, which encodes an L-type calcium channel subunit, is tightly regulated during cardiac development and is crucial for cardiac electrophysiological maturation [176]. In addition, two of the six pathways affected by these methylation changes were related to heart function [175]. This study was able to distinguish healthy and CHD infants of diabetic mothers based on DNA methylation patterns in buccal swab endothelial cells. Future studies are needed to determine whether methylation signatures could also distinguish between CHD patients of healthy mothers from those who were exposed to hyperglycemia in utero.

Using a diabetic mouse model, one study showed that altered chromatin accessibility in the setting of maternal hyperglycemia can influence critical cardiac developmental pathways [177]. Decreased chromatin accessibility associated with hyperglycemia caused an upregulation of *Jarid2*, a known epigenetic repressor of Notch1 signaling [177]. In contrast with the *Jarid2* knockout studies that observed CHD due to *Notch1* overexpression [80,118], this study found that the repression of *Notch1*, caused by maternal diabetes, contributed to the CHD phenotype. Environmental factors, including maternal diabetes, can perturb the epigenetic mechanisms that regulate gene dosing throughout cardiac development. Another study sought to determine whether factors other than hyperglycemia were involved in CHD associated with maternal diabetes. They were able to isolate circulating exosomes in the blood from diabetic maternal mice and identified significant differences in exosomal miRNAs compared to control mice. They confirmed that these exosomes can cross the placental barrier and infiltrate the embryonic heart. Interestingly, injecting these miRNAs into healthy mice increased the risk of CHD in the offspring [178]. Additional studies are required to determine how the differentially expressed exosomal miRNAs contribute to the cardiac defects associated with maternal diabetes.

## 6. Discussion

The prevalence and severity of CHD have warranted extensive research; however, the treatment of CHD is primarily palliative, and has remained largely unchanged for decades. Two principal factors contribute to this slow pace of discovery. First, heart development occurs early in embryogenesis as a series of complex morphogenic events involving at least four distinct cell sources. As a result, the cases of CHD that are detected before birth are usually detected well after the heart is malformed. Second, genome-wide analysis has identified distinct pathogenic mutations in only ~10% of cases [10,11,12,13,14,15,16,17,18]. While pathogenic gene mutations are likely involved in the remaining cases, epigenetics and mechanobiology may also cause CHD and certainly exacerbate defects and lead to pathogenic cardiac remodeling. To advance the diagnosis, understanding, and treatment of CHD, an improved understanding of these extra-genomic systems is necessary. Aberrant DNA methylation, histone modification, and miRNA expression have been observed in several types of human CHD, including TOF, HLHS, AVS, DORV, and VSD. These epigenetic processes regulate multiple stages of cardiac development, including cardiomyocyte differentiation and proliferation, chamber morphogenesis, valve formation, and septation. Hemodynamics and contractility are also necessary for cardiac development, playing roles in cardiac looping, valve formation, and myocardium trabeculation, compaction, and thickening. These intracardiac forces can alter gene expression by causing epigenetic modifications. Epigenetics and mechanotransduction tightly control the expression and activity of numerous cardiac proteins that yield severe CHD phenotypes in knockout studies.

While excellent progress has been made toward the understanding of epigenetic and mechanosensitive pathways involved in heart development, further investigation is needed to determine the causality of these pathways in CHD pathogenesis (Figure 4). Several studies have identified aberrant DNA methylation, histone modification, or miRNAs in biopsies from diseased tissues, but it is unclear whether these dysregulated epigenetic mechanisms caused the original defect or were altered as a result. It is likely that both explanations are at play. Given the former case, further investigation is required to determine the precise cause of the abnormal epigenetic control that leads to the formation of heart defects. Given the latter case, additional studies are needed to determine if epigenetic pathways are affected by the irregular mechanical forces that accompany structural heart defects. For example, the expression of miR-99, miR-100, and miR-145 returned to normal after palliative surgery and reduced volumetric loading in HLHS patients [109]. This result suggests that the overexpression of these miRNAs was due to abnormal mechanics caused by the malformed heart. To answer these questions, two approaches could be useful. First, integrating data collected from the genome, DNA methylome, histone marks, transcriptome, and prenatal environment (such as exposure to maternal diabetes) of CHD patients is likely to elucidate patient-specific etiology. Second, in vitro models using patient-derived induced pluripotent stem cells (iPSC) will be helpful for studying isolated biomechanical forces that are present during cardiogenesis on individual cardiac cell types. These models will also be useful for determining the causality of aberrant epigenetics in CHD, as epigenetic systems in specific cell types can be analyzed at different stages of cell differentiation or under distinct mechanical stimuli.

Clinically, the evaluation and modulation of epigenetics and mechanobiology for CHD diagnosis and treatment is extremely promising. HDAC inhibitors are currently being translated from oncology applications to adult heart failure and need to be investigated for use in cases of CHD. “Anti-miRs" and miRNA mimics are also being investigated as therapeutics for a wide variety of diseases due to their tissue specificity, and they have the potential to attenuate the formation of heart defects or pathologic remodeling characteristic of severe CHD. miRNAs and DNA methylation are also promising biomarkers for the prenatal and postnatal diagnosis and stratification of patients with CHD. Furthermore, both epigenetics and mechanobiology play critical roles in cardiomyocyte proliferation and may be useful for cardiac regenerative medicine in both pediatric and adult heart disease patients. Continued investigation of the distinct and overlapping roles of epigenetics and mechanobiology in heart development and CHD is essential for advancing our understanding of CHD and is likely to reveal new diagnostic and therapeutic strategies for the entire spectrum of heart diseases.

## Figures and Tables

**Table 1:** Epigenetic modifiers required for heart development.

Modifier	Modification	Target Gene(s)	Disease Phenotype	Ref.
**DNA Methylation**				
DNMT3B	Hypermethylation	*HAS2*	-	[73]
-	Hypermethylation	*APOA5, PCSK9*	AVS	[75]
-	Hypermethylation	*SCO2*	TOF, VSD	[76]
-	Hypermethylation	NKX2.5 *, HAND1	TOF	[77]
-	Hypermethylation	*ZIC3, NR2F2*	DORV	[78]
lncRNA uc.167	Hypermethylation	*MEF2C*	VSD, increased CM apoptosis	[74]
**Histone Modifications**				
Whsc1 *	H3K36me3	↓ NKX2.5 * target genes	ASD, VSD, Wolf-Hirschhorn Syndrome	[79]
JARID2/SETDB1	H3K9me3	↑ *NOTCH1* *	DORV, LVNC, VSD	[80]
HDAC2 *	Deacetylation	↓ GATA4 * target genes	Impaired cardiac differentiation and proliferation	[81]
HDAC3 *	Deacetylation	↓ TBX5 * target genes	Impaired cardiac differentiation	[82]
G9a/HDAC5	H3K9me2, HDAC5 association	↓ MEF2A/KLF2A target genes	Sarcomere disorganization, Valve malformation	[83,84]
SMyD1 *	H3K4me3	↑ SkNAC target loci	HRHS, CM hypoplasia	[85,86,87]
Ezh2/PRC2	H3K27me3, PRC1 recruitment	*TBX2, HEY2*	VSD, ASD, impaired endocardium, trabeculae, compaction, proliferation	[88,89]
-	↑ H3K27me3, ↓ H3K4me2	NKX2.5 *, HAND1, NOTCH1 *, TBX2, HEY	HLHS	[90]
Jmjd3	H3K27 demethylation	↑ *GATA4* *, *T*, *CTNNB1*	Impaired mesoderm/cardiac differentiation	[91]
UTX	H3K27 demethylation	↑ *GATA4* *, *NKX2.5* *, *TBX5* *	Arrested cardiac development after looping	[92]
MLL2 *	H3K4me3	↑ NKX2.5 *, TBX5 *, MEF2C	Abolished cardiac differentiation from mesoderm	[93,94]
Baf60c/Brg1	ATP-dependent histone-DNA destabilization	↑ *GATA4, TBX5, NKX2.5*	Impaired cardiac differentiation, OFT, and chamber formation	[95,96]
TBX1 *	Enhances Baf60a CRC H3K4me	↑ *WNT5A*	DORV, DiGeorge syndrome, HRHS, OFT defects	[97]
DPF3	Recruits BAF CRC to H3 and H4 methylation/acetylation sites	↑ *MEF2A*	Impaired looping and contractility	[98]
**miRNA**				
miR-1	Suppression	*HAND2, GJA1, SRF, MEF2, SOX9, HDAC4*	TOF, VSD	[99,100,101,102,103]
miR-17~92	Suppression	*ISL-1, TBX1, GJA1, FOG2*	Cardiac progenitor differentiation, VSD, angiogenesis	[104,105]
miR-21	Flow-dependent knockdown	*PDCD4, PTENB, SPRY2*	Valve and endocardial malformation	[106]
miR-23	Flow-dependent knockdown	*HAS2*	Valve and endocardial malformation	[107]
miR-137b/miR-204	Suppression	GATA4 *, HAND2	HLHS, pIDC	[108,109]
miR-99/miR-100/miR-145	Ventricular loading-dependent knockdown	*QKI, CDK6, SOX11, BAZ2A, FOG2, GATA6*	HLHS	[109]
miR-30/miR-133	Suppression	*CTGF, SRF MEF2A*	Impaired proliferation, cardiac fibrosis	[110]
miR-433	Suppression	NOTCH1 *, GATA3	VSD, RV dysfunction	[111]
miR-222	Suppression	*ZFPM-2*	TOF, RV morphogenesis, CM proliferation	[112]
miR-421	Suppression	*SOX2*	TOF	[113]
miR-940	Suppression	*JARID2*	TOF, impaired cardiac progenitor proliferation/migration	[114]
miR-143	Flow-dependent knockdown	*RALDH2, RXRAB*	Chamber and OFT development	[115]

* Gene mutations identified in human cases of congenital heart disease (CHD). Abbreviations: AVS (aortic valve stenosis), TOF (tetralogy of Fallot), VSD (ventricular septal defect), ASD (atrial septal defect), DORV (double outlet right ventricle), lncRNA (long non-coding RNA), CM (cardiomyocyte), HRHS (hypoplastic right heart syndrome), HLHS (hypoplastic left heart syndrome), pIDC (pediatric idiopathic dilated cardiomyopathy), OFT (outflow tract), RV (right ventricle), ↑ (upregulation), ↓ (downregulation).
